# Assessment of incidence and various demographic risk factors of traumatic humeral shaft fractures in China

**DOI:** 10.1038/s41598-018-38035-y

**Published:** 2019-02-13

**Authors:** Chenni Ji, Jia Li, Yanbin Zhu, Song Liu, Lei Fu, Wei Chen, Yingze Zhang

**Affiliations:** 1 Department of Orthopaedic Surgery, the Third Hospital of Hebei Medical University, Shijiazhuang, Hebei 050051 P. R. China; 2 Key laboratory of biomechanics of Hebei Province, Shijiazhuang, Hebei 050051 P. R. China; 3 Chinese Academy of Engineering, Beijing, 100088 P. R. China

## Abstract

As a sub-study of the China National Fracture Study, this study aimed to better determine the incidence and risk factors of humeral shaft fracture in mainland China. We obtained all the data on humeral shaft fracture from the China National Fracture Study reported in 2017. Trained research teams personally interviewed all qualifying household members using a standardised questionnaire. A total of 512,187 (259649 boys and men, 252538 girls and women) questionnaires were collected and analysed from 112 neighbourhood communities and 223 administrative villages using stratified random sampling and the probability proportional to size method. The population-weighted incidence rate of humeral shaft fracture was 7.22 (95% confidence interval 4.90, 9.55) per 100,000 populations in 2014. Previous fracture history was an independent risk factor in adults of both sexes. Smoking was identified as an independent risk factor for humeral shaft fracture for men. Alcohol consumption and menstruation ceasing before the age of 46 years were considered as independent risk factors for women. Given the above data, specific public-health policies focusing on promoting a smoke-free environment and reducing alcohol intake should be encouraged. People who have had a fracture and women whose menstruation had ceased before the age of 46 should be vigilant against humeral shaft fracture.

## Introduction

**Figure 1:**
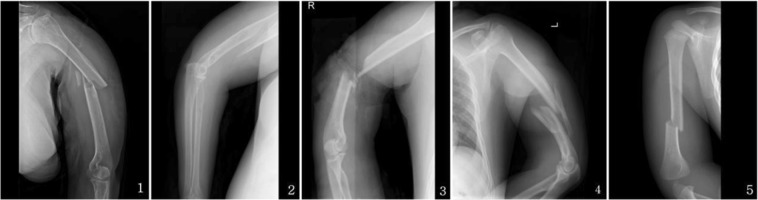
(1) Sample humeral shaft fracture. Female, 69 years old, osteoporosis, being knocked down by bicycle when walking in sidewalk. (2) Wedge humeral shaft fracture. Female, 64 years old, osteoporosis, falling down from bed. (3) Complex humeral shaft fracture. Male, 56 years old, falling from heights. (4) Complex humeral shaft fracture. Male, 28 years old, suffering motorcycle accident. (5) Sample humeral shaft fracture. Boy, 2 years old, slipping down when chasing and running.

Compared with hip and vertebral fractures, humeral shaft fracture is a much less frequent injury, which lacks sufficient consideration and epidemiological research in China. Traumatic humeral shaft fracture accounted for 1.17% of all fractures and up to 23.19% of all humeral fractures in 2010 and 2011^1^. Nevertheless, traumatic humeral shaft fracture frequently conferred an increased risk of limb deformity and may be associated with disability in some patients. This can result in severely reduced quality of daily activities and additional consumption of medical resources^2^. X-rays of simple, wedge and complex humeral shaft fractures are shown in Fig. 1.

Most previous studies of humeral shaft fracture considered inpatients only and focused on clinical management options^3^ or biomechanical evaluation^4^. In some published epidemiological studies^5–7^, the key points were restricted to prevalence and concomitant injury. The results^5–7^ might be limited by small sample size and restricted geographic areas. A large number of outpatients with fractures remains an underestimated but substantial burden on the health system. However, we did not record or study information regarding these patients. Furthermore, to formulate strategies for the prevention and management of traumatic fractures, information is needed from more than just admitted patients. Compared to other investigative approaches using questionnaires, in a face-to-face field investigation it is possible to obtain more reliable and precise information.

**Figure 2:**
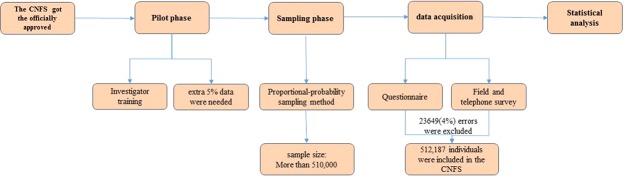
The design of the CNFS.

Given the above points, we designed the China National Fracture Study (CNFS) in 2015, from which data on risk factors and incidence of whole-body fracture stratified by age and gender were recently published^8^. Each skeletal fracture site is known to have its own specific characteristics, including incidence rate and related risk factors based on numerous parameters. However, this information was not reported in our previous article^8^. The design of the CNFS is presented in Fig. 2. Using the data extracted from the database of the CNFS, we conducted this study with the aim of better determining the incidence rate of humeral shaft fracture and exploring the associated risk factors in terms of demographic, lifestyle and socio-economic variables.

## Results

We identified a total of 37 (6 juvenile males, 19 males and 12 females) patients with traumatic humeral shaft fractures with a mean age of 42.9 years (standard deviation, 20.2 years; range, 6–72 years). The incidence rate of traumatic humeral shaft fractures in China was 7.22 (95% confidence interval (CI) 4.90–9.55) per 100,000 populations, with 9.63 (95% CI 5.85–13.4) for males, and 4.75 (95% CI 2.06–7.44) for females. We first analysed the incidences by individual characteristics and regions (Table 1). There were no significant differences in incidence in subgroups of ethnic origin, urbanisation, occupation or education. We found the highest incidence rates in the 5–14, 55–64, and 65–74 age groups. These were 11.04 (95% CI 2.21, 19.88), 20.35 (95% CI 8.84, 31.86) and 10.32 (95% CI 0.21, 20.44) per 100,000, respectively. In males stratified by age, the 5–14, 55–64, and 65–74 age groups had the highest incidence of 20.30 (95% CI 4.06, 36.55), 17.30 (95% CI 2.14, 32.47), and 10.35 (95% CI −3.99, 24.70) per 100,000, respectively. In females, the three highest incidence rates were 23.28 (95% CI 6.03, 40.52), 10.30 (95% CI −3.97, 24.57), and 7.63 (95% CI −1.00, 16.26) per 100,000 in the 55–64, 65–74, and 45–54 age groups, respectively. There was a significant difference in the incidence rate among the age groups in women (P < 0.001) and the combined sexes (P = 0.009). Regarding the geographical regions, we found the highest incidence rate of 11.11 (95% CI 6.24–15.97) per 100,000 (P = 0.011) in the west. The incidence of humeral shaft fracture of 16.59 (95% CI 8.19–24.98) per 100,000 in males from the western region was the highest of the three regions. There was a significant difference in the incidence rate among the western, eastern and central regions (P = 0.008).

**Table 1:** National incidence of traumatic humeral shaft fractures among the Chinese population by demographic, socio-economic and geographic factors in 2014.

Items	Sample size	Incidence rate per 100 000 people (95% CI)		
		Male	Female	Total
**Total**	512,187	9.63 (5.85, 13.4)	4.75 (2.06, 7.44)	7.22 (4.90, 9.55)
Age (years)				
0–4	26,840	0	0	0
5–14	54,326	20.30 (4.06, 36.55)	0	11.04 (2.21, 19.88)
15–24	62,020	9.65 (−1.27, 20.57)	0	4.84 (−0.64, 10.31)
25–34	93,194	8.69 (0.17, 17.20)	0	4.29 (0.09, 8.50)
35–44	80,992	7.31 (−0.96, 15.59)	0	3.70 (−0.50, 7.90)
45–54	79,565	4.97 (−1.92, 11.86)	7.63 (−1.00, 16.26)	6.28 (0.77, 11.79)
55–64	58,968	17.30 (2.14, 32.47)	23.28 (6.03, 40.52)	20.35 (8.84, 31.86)
65–74	38,745	10.35 (−3.99, 24.70)	10.30 (−3.97, 24.57)	10.32 (0.21, 20.44)
75+	17,537	0	0	0
*P*-value for difference test	512,187	0.464	<0.001	0.009
Ethnicity				
Han nationality	477,508	7.33 (4.90, 9.76)	9.50 (5.62, 13.39)	2.10 (2.21, 7.98)
Other nationalities	34,679	5.77 (−2.23, 13.76)	11.36 (−4.38, 27.09)	0
*P*-value for difference test	512,187	0.809	0.351	0.741
Region				
East	232,998	8.38 (3.18, 13.57)	5.28 (1.06, 9.51)	6.87 (3.50, 10.23)
Central	99,109	0	2.03 (−1.95, 6.00)	1.01 (−0.97, 2.99)
West	180,080	16.59 (8.19, 24.98)	5.58 (0.69, 10.47)	11.11 (6.24, 15.97)
*P*-value for difference test	512,187	0.008	0.617	0.011
Urbanisation				
Urban area	203,101	7.39 (3.65, 11.12)	8.77 (3.04, 14.50)	5.97 (1.19, 10.75)
Rural area	309,086	7.12 (4.14, 10.09)	10.19 (5.20, 15.18)	3.95 (0.79, 7.10)
*P*-value for difference test	512,187	0.718	0.469	0.902
Occupation				
Office worker	61,919	6.10 (−2.35, 14.56)	3.43 (−3.29, 10.16)	4.85 (−0.64, 10.33)
Farmer	106,484	8.21 (0.16, 16.25)	6.92 (0.14, 13.71)	7.51 (2.31, 12.72)
Manual worker	148,650	8.46 (2.19, 14.73)	4.55 (−0.60, 9.70)	6.73 (2.56, 10.90)
Retired	30,366	6.74 (−6.47, 19.94)	6.44 (−6.19, 19.07)	6.59 (−2.54, 15.71)
Unemployed	32,770	41.38 (0.84, 81.93)	12.98 (−1.71, 27.68)	21.36 (5.54, 37.18)
Preschool Children	35,581	0	0	0
Students	80,443	14.17 (2.83, 25.51)	0	7.46 (1.49, 13.43)
Other	15,974	11.04 (−10.60, 32.67)	0	6.26 (−6.01, 18.53)
*P*-value for difference test	512,187	0.057	0.431	0.093
Education (Preschool children and students were excluded)				
Illiterate	74,937	14.51 (1.79, 27.23)	12.35 (1.53, 23.18)	13.34 (5.07, 21.62)
Primary school	158,970	11.21 (3.89–18.54)	6.35 (0.78–11.92)	8.81 (4.19, 13.42)
Junior high school	121,415	4.88 (−0.64, 10.40)	3.34 (−1.29, 7.96)	4.12 (3.30, 4.76)
Senior high school or above	40,841	9.26 (−3.57, 22.10)	0	4.90 (−1.89, 11.68)
*P*-value for trend test	396,163	0.473	0.207	0.128

The proportions of each category of causal mechanism for humeral shaft fracture in juveniles, young and middle-aged adults, and older adults are summarised in Table 2. A slip, trip or fall was the most frequent cause of injuries (19, 51.35%). This accounted for 83.33% of injuries in juveniles, 41.18% in young and middle-aged men, 50.00% in young and middle-aged women and 100% in older women. Traffic accidents (10, 27.03%) were the second most common cause of injury in all subpopulations, except in older women. Crushing injuries (6, 16.22%) were the third most frequent cause of injury in the young and middle-aged group, and in older men. Other injuries were caused by falls from height (1, 2.7%) and blunt-force trauma (1, 2.7%). Most of the humeral shaft fractures either occurred at home or were associated with traffic accidents (32, 86.49%).

**Table 2:** Proportion of traumatic humeral shaft fractures by causal mechanisms in China in 2014.

Injury Mechanism	Juveniles (0–14 years)	Young and middle-aged (15–64 years)		Elderly population (≥65 years)		Total
		Male	Female	Male	Female	
Traffic Accident	16.67 (−13.15, 46.49)	29.41 (7.75, 51.07)	30.00 (1.60, 58.40)	50.00 (−19.30, 119.30)	..	27.03 (12.72, 41.34)
Slip, Trip or Fall	83.33 (53.51, 113.15)	41.18 (17.78, 64.57)	50.00 (19.01, 80.99)	..	100.00 (100.00, 100.00)	51.35 (35.25, 67.46)
Fall from Heights	..	..	10.00 (−8.59, 28.59)	..	..	2.70 (−2.52, 7.93)
Crushing Injury	..	23.53 (3.67, 43.69)	10.00 (−8.59, 28.59)	50.00 (−19.30, 119.30)	..	16.22 (4.34, 28.09)
Sharp Trauma	..	..	..	..	..	..
Blunt Force Trauma	..	5.88 (−5.30, 17.07)	..	..	..	2.70 (−2.52, 7.93)

Data are % (95% confidence interval). Double dots indicate no fracture cases recorded in this subgroup.

We performed a univariate logistic analysis for all adults (≥15 years) (Table 3). In men, cigarette smoking (P = 0.002) and a previous history of fracture (P = 0.003) were significant risk factors for humeral shaft fractures. In women, alcohol consumption (P = 0.001), a previous history of fracture (P = 0.002) and menstruation ceasing before the age of 46 (P = 0.002) were identified to have a significant effect on the occurrence of humeral shaft fractures. We analysed risk factors for traumatic humeral shaft fractures in adults aged >15 years by sex in a multivariate model and these are summarised in Table 4. Cigarette smoking was an independent risk factor in males with an adjusted odd ratio (OR) of 9.50 (95% CI 2.19, 41.19). Alcohol consumption was an independent risk factor in females with an adjusted OR of 7.81 (95% CI 2.50, 24.39). In both males and females, a previous history of fracture was a strong risk factor associated with traumatic humeral shaft fractures, with an adjusted OR of 5.30 (95% CI 1.54, 18.23) and 6.77 (95% CI 1.47, 31.19), respectively, after eliminating the effects of confounding factors. Menstruation ceasing before the age of 46 years was a risk factor in women. The results of the Hosmer-Lemeshow test demonstrated a good fit with *X*^2^ = 0.280, P = 0.964 for males and *X*^2^ = 4.174, P = 0.525 for females (Table 4).

**Table 3:** Univariate analysis of factors associated with humeral shaft fractures in adults.

Variables	Number of individuals without humeral shaft fractures		Number of humeral shaft fractures		*P*	
	Male (n = 215397)	Female (n = 215593)	Male (n = 19)	Female (n = 12)	Male	Female
Age groups (years)						
15–59	170990	170873	14	8	0.541	0.290
≥60	44407	44720	5	4		
BMI						
<24	148554	161163	12	7	0.572	0.203
24–27.9	58445	45004	6	4		
≥28	8398	9426	1	1		
Education						
Illiteracy	34592	40604	5	5	0.082	0.028
Primary school	82734	80882	9	5		
Junior school or above	98071	94107	5	2		
Occupation						
Unemployed and Retired	24507	38622	5	2	0.439	0.639
Office worker and Student	50389	46414	2	1		
Others	9047	6904	1	2		
Manual worker and Farmer	131454	123653	11	7		
Cigarette smoking						
No	117224	208487	2	12	0.002	0.981
Yes	98173	7106	17	0		
Calcium or VD supplement						
No	204439	201377	17	12	0.293	0.973
Yes	10958	14216	2	0		
Alcohol consumption						
No	100992	189119	5	6	0.082	0.001
Yes	114405	26474	14	6		
Average sleep time per day(h)						
≥7	73623	76456	10	6	0.098	0.300
<7	141774	139137	9	6		
Previous history of fracture						
No	209398	211745	16	10	0.003	0.002
Yes	5999	3848	3	2		
Urbanisation						
Urban area	85442	85974	8	6	0.828	0.477
Rural area	129955	129619	11	6		
Children						
No or only one		115878		2		0.144
2		68942		9		
>2		30773		1		
Menopause age(years)						
<46		5392		1		0.002
46–50		57674		7		
>50		19421		3		
Premenopausal		133106		1

**Table 4:** Risk factors for traumatic fractures among Chinese adults (≥15 years).

Risk factors	Odds ratio (95% CI)	
	Males	Females
Age (years)		
15–59	Reference	Reference
≥60	0.56 (0.15, 2.03)	0.69 (0.17, 2.76)
Education		
Illiterate	Reference	Reference
Primary school	0.84 (0.27, 2.64)	0.80 (0.22, 2.90)
Junior high school or above	0.50 (0.14, 1.82)	0.40 (0.07, 2.15)
Occupation		
Unemployed & Retired	Reference	Reference
Office worker & Students	0.22 (0.04, 1.35)	1.47 (0.12, 17.80)
Others	0.39 (0.04, 3.62)	4.30 (0.59, 31.41)
Manual worker & Farmer	0.29 (0.08, 1.00)	1.43 (0.27, 7.43)
Cigarette smoking		
No	Reference	
Yes	9.50 (2.19, 41.19)	
Alcohol consumption		
No	Reference	Reference
Yes	1.49 (0.52, 4.25)	7.81 (2.50, 24.39)
Urbanisation		
Urban area	Reference	Reference
Rural area	0.76 (0.30, 1.93)	0.64 (0.20, 2.07)
Body Mass Index (BMI)		
<24	Reference	Reference
24–27.9	1.15 (0.43, 3.06)	1.17 (0.34, 4.02)
≥28	1.24 (0.16, 9.56)	1.10 (0.13, 9.08)
Average sleep time (hours) per day		
<7	Reference	Reference
≥7	0.54 (0.21, 1.38)	0.90 (0.28, 2.91)
Previous history of fracture		
No	Reference	Reference
Yes	5.30 (1.54, 18.23)	6.77 (1.47, 31.19)
Children		
<2	/	Reference
=2	/	3.60 (0.75, 17.17)
>2	/	0.65 (0.06, 7.55)
Menopause (age, year)		
<46	/	Reference
46–50	/	0.75 (0.09, 6.12)
>50	/	1.03 (0.11, 9.98)
Premenopausal	/	0.06 (0.003, 0.941)
Hosmer-Lemeshow Test	*X*^2^ = 0.280, *P* = 0.964	*X*^2^ = 4.174, *P* = 0.525

## Discussion

The epidemiological features of the humeral shaft fracture have been reported in Europe^9–12^, the USA^13,14^, Africa^15,16^ and the Taiwan province of China^2^. Annual incidence rates of humeral shaft fractures in some European countries, the USA and China are summarised in Table 5. Findings from the current study showed that the annual population-weighted incidence of humeral shaft fracture in mainland China of 7.22 per 100,000 was lower than that in the USA^13,14^, Europe^10–12^ and Taiwan province^2^. Most studies reported a bimodal age distribution of humeral shaft fractures^2,10,11^, while another found that the majority of humeral shaft fractures occurred among those aged <35 years^17^. A bimodal age-specific distribution has been delineated both in Taiwan^2^ and in the mainland. There were two peaks in annual incidence rate for humeral shaft fracture, occurring in the third decade, resulting from high-energy trauma, mainly in men^2^, and in the eighth decade, mostly in women with osteoporotic fractures resulting from simple falls^2,9–11,18^. Our results showed that two peaks occurred, at 5–14 years, mainly in male juveniles, and at 55–64 years in adults, with approximately similar incidences in both sexes. Qiling *et al*.^19^ suggested that juveniles of 5–14 years performed considerably more physical activities than younger juveniles (<5 years), and, therefore, were more likely to be exposed to danger. Moreover, safety awareness in children was increased with greater education and life experience^20^. The older age group were more self-aware regarding personal safety than the younger age group^21^. In the present study, the results showed greater activity and traffic injuries resulted in a higher incidence of humeral shaft fracture in the 5–14 age group. In addition, we observed that the peak value of the incidence rate of humeral shaft fracture was in the 55–64 age group, being higher for women than for men. Previous studies demonstrated that humeral shaft fracture in older women is closely related to osteoporotic fractures^2,9,11,17,18^. Menopause has a significant association with osteoporosis, and a higher risk of fragility fracture generally results from reduced oestrogen levels, which lead to declining bone mass density^22–25^. Trémollieres *et al*.^26^ used the FRAX tool to evaluate the 10-year fracture incidence rate in early postmenopausal women and found that those in their 3rd or 4th year after menopause had the greatest risk for major osteoporotic fractures. Huang *et al*.^27^ and Li *et al*.^28^ suggested that the bone loss rate within the first 5 years after menopause was the most rapid (5.31–9.44%), which was 1.1–1.6 times and 2.0–3.0 times that occurring during the 6–10 years and the subsequent 20 years after menopause, respectively. The Court-Brown study^12^ demonstrated a relatively uniform incidence of fracture in women up to the time of menopause and a rapid increase thereafter. It was suggested that the marked bone loss occurred because of dramatically declining oestrogen levels in the first 3–4 years of menopause^29^. Meanwhile, the maximum synergistic effects exerted by declining oestrogen levels, ageing, and other comorbidities^29^ may have facilitated the peak incidence rate of humeral shaft fracture in the 55–64 age group. Related to these points, the finding of the current study showed that women whose menstruation had ceased before the age of 46 displayed an increased risk for humeral shaft fracture compared with all those who were in premenopausal.

**Table 5:** Annual incidence rates of humeral shaft fractures (per 100,000) in other countries.

Author	Year [ref.]	Origin of study	Date year	Age of patients	Sex	Annual rate
Ekholm, R. *et al*.	2006^11^	Sweden	1998–1999	≥16 years	Both	14.5
					Male	17
					Female	20
Court-Brown, C. M. *et al*.	2006^12^	Edinburgh, UK	2000	≥12 years	Both	12.9
Tsai, C. H. *et al*.	2009^2^	Taiwan, China	2003–2007	>0	Both	10
Kim, S. H. *et al*.	2012^14^	US	2008	>0	Both	19
In current study	—	Mainland, China	2014	>0	Both	7.22
				≥15 years	Male	8.82
				≥15 years	Female	5.57
				<15 years	Both	7.39

The humeral shaft fracture incidence rate in western populations was significantly higher than for those living in the east (P = 0.011), but this trend only appeared significant for males (P = 0.008), and not for females (P = 0.617). In China, economic development is relatively different between urban and rural areas and among different regions, with the per-capita GDP of some inland provinces being only 33% of that of the coastal region. Because of the slower social-economic development of the western region compared with the eastern region, much of the employment in the west, including heavy industry, construction, and transportation, carries a higher risk of injury^30^. Second, according to the China Statistical Yearbook 2014^31^, the proportion of the population aged >65 years is 10.5% in the west and 9.8% in the east. An ageing population and unequal regional development contributed to the higher incidence of humeral shaft fracture in the western region.

High-energy trauma caused by traffic accidents was a significantly more frequent cause of humeral shaft fractures occurring in Taiwan (63.2%)^2^. By contrast, our results showed that 51.35% of humeral shaft fractures resulted from a slip, trip or fall, and only 27.03% from traffic accidents. We postulate that the difference between the studies may depend on economic, ageing and lifestyle factors. Because of the combination of the law against drunk driving and speed restrictions, the occurrence of traffic injuries remained low^32^. It has been previously reported that the incidence of humeral shaft fractures continually increased within an ageing population^3,4,14^. A European study^12^ showed the same distribution of causes of fracture in older women with ours. Combined with menopause, the occurrence of low-energy injuries, for example, from a simple fall, increases because of osteoporosis^25^. Consequently, the age-specific incidence of humeral shaft fractures in women over 45 years gradually increased in the present study. In the current study, cigarette smoking in men and alcohol consumption in women were considered independent risk factors for humeral shaft fractures for populations aged ≥15 years. It has been reported that smoking has a negative influence on bone mineral density, which increases the risk for fractures^33^. Cornuz *et al*.^34^ reported an increased relative risk of 1.3 (95% CI 1.0–1.7) for hip fracture in current smokers compared with non-smokers. Furthermore, a reduced relative risk of 0.7 (95% CI 0.5–0.9) demonstrated that having quit smoking for more than 10 years was beneficial to fracture prophylaxis^34^. Much greater education and smoking control regulations are needed. According to previous literature^32,35^, alcohol consumption had more negative effects on women with the underlying mechanisms of metabolic disorders and alcohol-related falls^32^. It was reported that a weekly consumption of more than eight units of alcohol by men and more than six units by women significantly increased the risk of fractures in individuals aged ≥55 years^32^. Our results indicated that alcohol consumption was a clear risk factor for traumatic humeral shaft fracture in women, but not in men. The authors suggest that the result in men might be a false negative. A close relationship between low-energy fracture and a history of previous fracture in middle-aged women^36^ and older men and women^37^ has been reported, which we also found in our study. We obtained similar findings that a history of previous fracture frequently predicted further fractures^25^, including humeral shaft fractures in both sexes when aged >14 years. Therefore, more education and intervention regarding the prevention of fractures should be promoted.

The strengths of the present study were as follows: (1) This is clearly the largest sample-size survey that we are aware of, with over 500,000 participants from eight provinces on the Chinese mainland, indicating a representative population-based epidemiologic study. (2) A stratified random-sampling method was used to recruit subjects. (3) A face-to-face interview was used to obtain precise information. However, there were some limitations to the study that need to be considered. First, data on subjects who died after fractures in 2014 were unavailable for the survey, which may result in a certain degree of selection bias. Second, because of the retrospective design, there is a possibility of data inaccuracy in the context of recall bias. Furthermore, the number of humeral shaft fracture cases appeared too low in some subgroups, which might be related to an inadequate statistical ability to find other potential risk factors.

## Conclusions

In summary, we have presented, for the first time, detailed epidemiologic characteristics of humeral shaft fractures in mainland China. Our study could serve as a basis for further clinical studies on prophylactic therapy and provide references or guidelines in establishing related national health planning and consultation. This could support promoting a smoke-free environment, decreased alcohol consumption and keeping vigilance against fracture again and factors related to premature menopause.

## Methods

This study is registered with the Chinese Clinical Trial Registry, number ChiCTR-EPR-15005878, reviewed and approved by the Institutional Review Board of the Third Hospital of Hebei Medical University. Written informed consent was obtained from all participants before data collection. All methods were performed in accordance with the relevant guidelines and regulations.

### Pilot phase of the CNFS

To estimate the general incidence of fracture within the Chinese population and facilitate more accurate sample-size planning based on the probability proportional to size sampling (PPS) method, we undertook a pilot phase. A total of 3,299 individuals were recruited from two urban communities and three administrative villages of Hebei Province. Following the PPS calculations, 510,000 individuals were set as the final target sample size to meet recommended requirements for precision in a complex survey design. Findings of the pilot phase showed that 3.2% of responses were unusable, and, therefore, we added an extra 5% to the total required sample size in both urban and rural areas for the main study. Therefore, more than 535,500 questionnaires were necessary.

### Sampling method and sample size of the CNFS

Eight provinces (municipalities) were chosen from three regions (three from the east, two central and three west), with a total of 31 provinces (municipalities or autonomous regions) of mainland China. The choices were based on geographical location, socio-economic development, climate and population size, by stratified random sampling. In each targeted province, we performed sampling separately in urban and rural areas.

In urban areas, we first assigned cities to three strata (large, medium-sized and small) by population size^38–40^. Then in each urban stratum, we selected representative cities by proceeding geographically from west to east using the PPS sampling method. The same methods were applied to select a certain number of targets ranging from 1–6 streets in each city chosen and 1–10 neighbourhood communities in every selected street. Finally, 24 cities, 41 streets and 112 neighbourhood communities were confirmed as the target sample. In each neighbourhood community, we determined the total number of selected families by the mean number of household members according to official census data and their apartment numbers^8^. In each neighbourhood community, 430 to 7,878 individuals were targeted, so we could reach the target sample of 201,696 subjects.

In rural areas, we sampled 1–5 counties in each selected province, 1–8 towns in each selected county and 1–14 villages in each selected town using the PPS sampling method. Households were assigned identification numbers based on official census records and PPS principles. The selected households were sequenced geographically from west to east and from north to south. Finally, 24 counties, 67 towns and 223 administrative villages were confirmed as the target sample. For each village chosen, 424 to 4,519 individuals were sampled, so we could meet our target number of 308,304 individuals from rural areas. All members of eligible families that were to be invited to participate in the study must have resided at their current address for a minimum of 6 months.

### Participants and survey

We established eight trained research teams and eight quality control teams, including independent orthopaedic surgeons and radiologists. Every team had been assigned to different province then completed the field survey between 19 January and 16 May 2015. All eligible household members were personally interviewed by trained research teams using a standardised questionnaire. Before the interview, we obtained a voluntarily signed written informed consent from every participant. Each quality control team sampled 10% of all questionnaires for omissions and errors to ensure the accuracy of the answers, particularly regarding the original diagnosis.

### Definition of variables of interest

We investigated various factors of interest, which have potential correlations with fractures, including age, sex, Chinese ethnic nationality, education, urbanisation, occupation, body mass index, and previous fracture history. We also investigated cigarette smoking, alcohol consumption, calcium or vitamin D supplementation and daily sleeping time, as well as the childbirth record and menstruation cessation age for women. Participants considered their status with regard to these factors before the fracture occurrence (for participants with fractures) or before answering the questionnaire (for those without fractures). The body mass index was calculated as weight divided by the square of height, with three subgroups: underweight and normal (<24), overweight (24–27.9), obesity (≥28). We defined calcium or vitamin D supplementation as positive when participants acknowledged that they had received calcium and/or vitamin D at least 1 month before the humeral shaft fracture occurrence or during the whole of 2014. We classified daily sleep into two groups: <7 hours or ≥7 hours. We classified education into three groups: illiteracy, primary school and junior school, or above. We classified occupation into four groups based on the degree of labour involved: unemployed or retired, office worker or student, manual worker or farmer, and others.

Participants who had experienced a fracture during 2014 were asked to provide extra medical records of their fractures, including fracture site, date and location of fracture occurrence and injury mechanism. We classified the injury mechanism into groups: traffic accident, sample falls (slip, trip, or fall), fall from a height, crushing injury, sharp trauma and blunt-force trauma. When such information was unavailable, a telephone consultation or a new radiograph of the reported fracture site would be performed.

### Statistical analysis

We analysed data using SPSS version 19.0 software (IBM Corp., Armonk, NY, USA). We estimated incidence rates for humeral shaft fractures for the overall populations and for subgroups, including age, region, ethnic origin, occupation and education, among others, all stratified by gender. For unordered categorical variables, including occupation, region, and ethnic origin, we applied the chi-square test to test the differences. We expressed differences in incidence between categorical variables as numeric values (percentages) and compared these using the chi-square (*X*^2^) test or Fisher exact test, as appropriate. We performed a univariate logistic analysis to evaluate the relationship between each categorical variable and humeral shaft fracture. The significance threshold was set at *P* < 0.05. All variables were entered into a multivariable logistic regression model to determine their independent effects on fracture occurrence. Values are expressed as the OR with their corresponding 95% CI and *P*-values. We used the Hosmer-Lemeshow test to examine the goodness of fit of this model, and a *P*-value > 0.05 indicated an acceptable level of fit.

## Data Availability

Most of the data generated or analysed during this study are included in this published article. All data generated during and analysed in the current study are available from the corresponding author on reasonable request.
